# Electrical storm refractory multiple antiarrhythmic medications was stopped by interatrial shunting procedure—A case report

**DOI:** 10.3389/fcvm.2022.1012916

**Published:** 2022-11-17

**Authors:** Caiping Han, Rujie Qiu, Lei Li, Min Han, Chengyi Xu, Li Liu, Chengwei Liu

**Affiliations:** Division of Cardiac Care Unit, Department of Cardiology, Wuhan Asia Heart Hospital, Wuhan, China

**Keywords:** electrical storm, antiarrhythmic medications, mechanical-electric feedback, left ventricular filling pressure, interatrial shunting

## Abstract

Electrical storm (ES) remains a major dilemma for clinicians, often presenting as a medical emergency associated with significant adverse outcomes. The mechanisms behind triggering ES are complex. Although the increased activation of the sympathetic nervous system was widely accepted as a major mechanism in initiating and maintaining ES, it's thought that the interaction between mechanical and electrical substrates may play an important role in some situations. Here we present a case of ES that was refractory to multiple antiarrhythmic medications but was stopped by interatrial shunting. We aim to highlight the importance of mechano-electric feedback (MEF) as the pathophysiological mechanisms of some types of ES and the utility of interatrial shunting as an alternative therapeutic strategy for patients with ES initially refractory to antiarrhythmic medications when there is evidence to indicate increased left ventricular filling pressure or left atrial pressure.

## Introduction

Electrical storm (ES) remains a major dilemma for clinicians, often presenting as a medical emergency associated with significant adverse outcomes. It is defined as three or more sustained episodes of ventricular tachycardia (VT)/ventricular fibrillation (VF) within 24 h, one or more episodes occurring within 5 min of termination of the previous episode of VT/VF (1, 2). It typically occurs in patients with underlying heart disease, such as structural heart diseases, inherited arrhythmia syndromes (e.g., channelopathies, Brugada syndrome, early repolarization, and premature ventricular contraction-induced ventricular fibrillation), prolonged QT, ischemic or non-ischemic cardiomyopathy, and electrolyte imbalances (1–3). Acute management often requires multimodality approaches including deep sedation, sympathetic blockade (using beta blockers), administration of anti-arrhythmic drugs, electrical cardioversion, interventional techniques (such as catheter ablation and neuraxial modulation), or insertion of an implantable cardioverter defibrillator (ICD), but the patients are not always responsive to these approaches (1–3). The precise mechanisms behind triggering ES are complex and still remain elusive. It's thought that the interaction between mechanical and electrical substrates may play an important role in some situations (4, 5). Acute mechanical stretch of the heart can depolarize the cell membrane and shorten the action potential duration by activating mechano-sensitive ion channels, inducing the onset of arrhythmias through the modulation of the myocardial electrophysiological properties (5, 6). Here we presented a case of ES that was refractory to multiple antiarrhythmic medications and repetitive electrical shock but was stopped by a procedure of interatrial shunting, suggesting the role of mechano-electric feedback (MEF) in triggering some types of ES. MEF refers to the mechanically-induced alterations in cardiac electrophysiology. In this report, we aim to highlight the importance of MEF in the pathophysiological mechanisms of some types of ES and the utility of this practice as an alternative therapeutic strategy for patients with ES initially refractory to antiarrhythmic medications.

## Case presentation

A 55-year-old man presented to our hospital with complaints of progressively worsening angina upon exertion, radiating to his back for the past 2 weeks. He reported accompanying diaphoresis and chills. His past medical history was significant for hypertension (HTN) for 15 years and type 2 diabetes mellitus (DM2) for 8 years, but he was not compliant with medications. He received a thoracic endovascular aneurysm repair (TEVAR) for an aortic dissection 12 years prior and a stent implantation at the mid-segment of the right coronary artery (RCA) at a local hospital 1 month ago due to unstable angina (UA).

**Figure 1:**
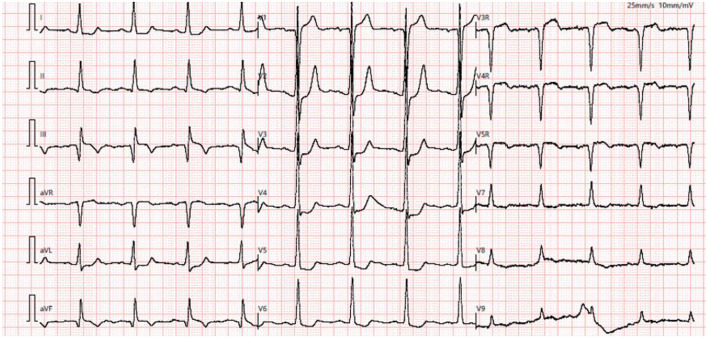
Baseline ECG showed sinus rhythm with mild ST-T elevation in aVR and V1 leads and ST-T depression in I, aVL, and V2–V5 leads.

At admission, a physical examination revealed a normal range of blood pressure (120/68 mmHg), heart rate (74 beats/min), respiratory rate (18/min), and oxygen saturation level at 99% (room air). Cardiac and pulmonary examination revealed normal heart sounds without murmurs and clear lungs. He denied smoking and drinking. Labwork indicated that blood glucose was elevated at 12.3 mmol/L, as well as initial troponin (17.12 ng/ml) and NT-proBNP (1186 pg/ml), but CBC w/ diff, CMP, renal function, and hepatic function were within normal limits. Baseline ECG displayed sinus rhythm (HR = 74 bpm) with likely “left main coronary artery lesion” (mild ST-T elevation in aVR and V1 leads and ST-T depression in other 6 leads) (Figure 1). Transthoracic echocardiogram (TTE) revealed mild enlargement of the left ventricular (LV) chamber (51 mm), low LV ejection fraction (LVEF, 40%), and mild mitral and tricuspid regurgitation. The diagnoses were as follows: coronary artery disease, acute myocardial infarction (AMI), post-percutaneous coronary intervention (PCI), hypertension, DM2, and post-TEVAR. The patient was transferred to the cardiac care unit (CCU) and managed with anti-hypertensive, anti-diabetic, anti-platelet, and lipid-lowering medications but his chest pain was not well-controlled.

**Figure 2:**
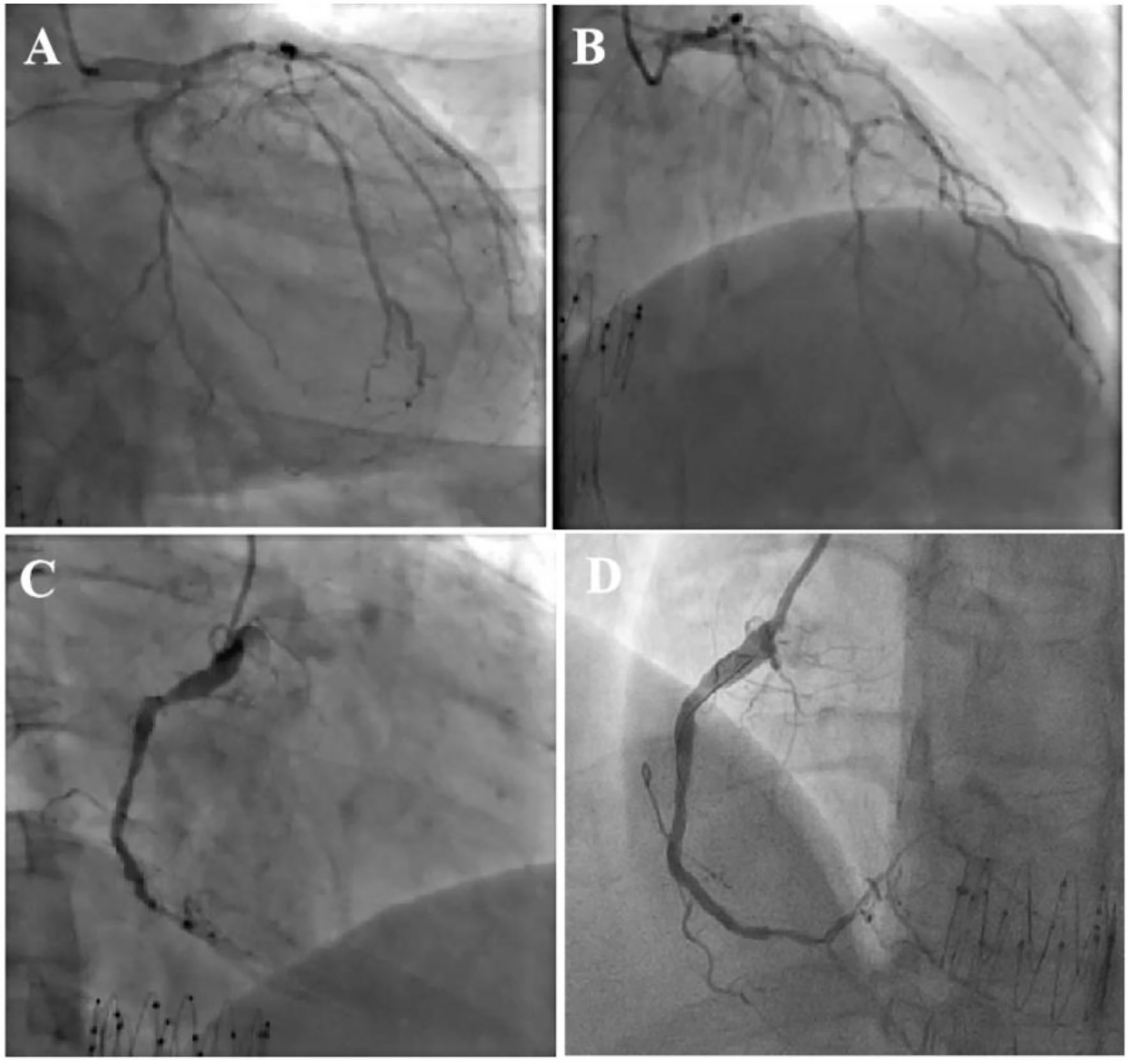
Coronary angiography showed severe three-vessel coronary artery disease with severely diseased and calcified left circumflex artery (LCX) **(A)** and left anterior descending artery (LAD) involved in multiple segments **(B)**. RCA angiography unveiled fluent stent and diffused lesions in distal segment, and subtotal occlusion close to post-trigeminal area **(C)**, the distal lesion of RCA was treated with drug-coated balloon with satisfactory result **(D)**.

On the eighth day of hospitalization, the patient underwent cardiac catheterization and trans-radial coronary angiography showed severe three-vessel coronary artery disease (CAD) with a severely diseased and calcified left circumflex artery (LCA, 80–90% stenosis) and left anterior descending artery (LAD, 90–95% stenosis) in multiple segments (Figures 2A,B and Supplementary Videos 1, 2). RCA angiography unveiled fluent stent in the second segment and diffuse lesions in the distal segment, with subtotal occlusion close to the post-trigeminal area (Figure 2C and Supplementary Video 3). The distal lesion of RCA was treated with a drug-coated balloon (Figure 2D and Supplementary Video 4). Intervention for LAD and LCA was planned in 3 days and the patient was sent back to CCU.

**Figure 3:**
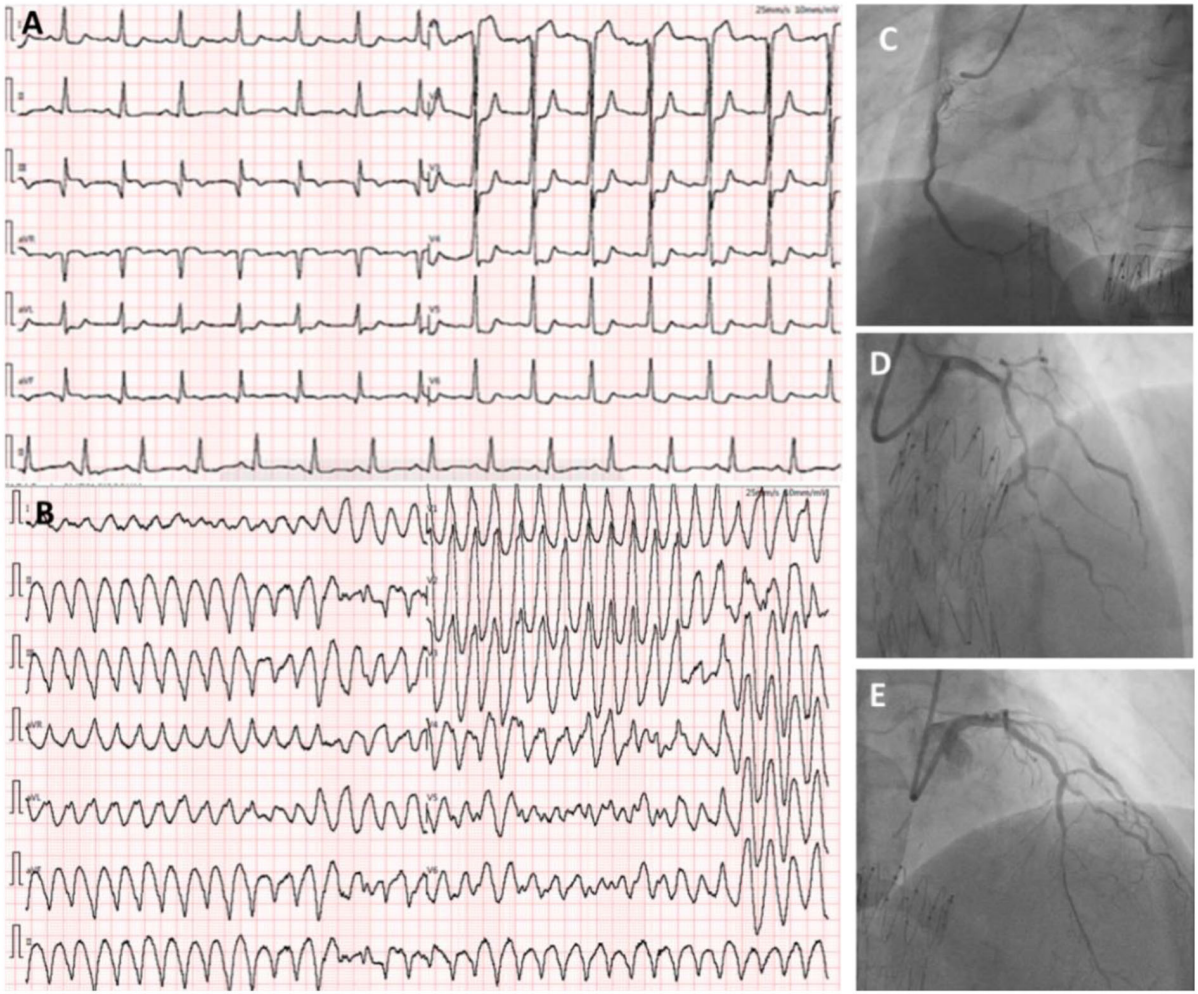
ECG showing ST-elevation in V1 and ST-depression in I, aVL, and V1–V6 leads when the patient complained of severe chest pain **(A)**. There is an ES attack during the reported chest pain **(B)**. Coronary angiography demonstrated patency of RCA (TIMI flow grade II-III) **(C)**. Three stents were implanted in the LAD with remarkable results (TIMI flow grade III) **(D,E)**.

**Figure 4:**
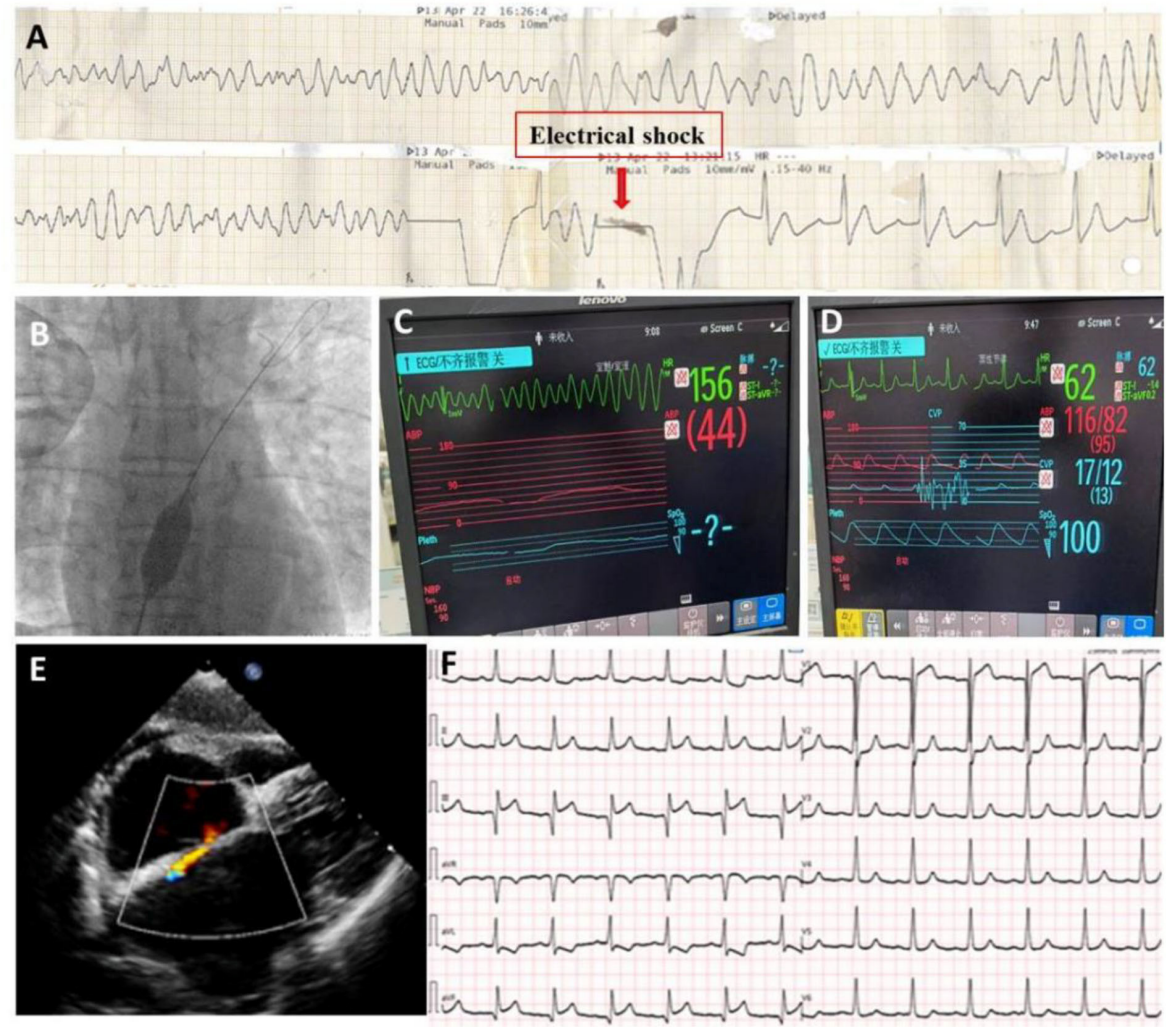
After stents implantation in LAD, patient still suffered from ES attack **(A)**; A procedure of interatrial shunting was performed **(B)**; The ES continues during procedure **(C)**; but stopped immediately after the creation of shunting **(D)**; TTE demonstrated s 9 mm interatrial shunting **(E)**. Follow-up ECG showed sinus rhythm except for a slight ST elevation in II, II, and aVF leads **(F)**.

However, on the morning of the second day after the RCA intervention, the patient complained of severe chest pain. ECG demonstrated diffuse ST-T changes in anterior leads (Figure 3A) and the patient suffered from an ES attack (repeated VF) soon after (Figure 3B). The patient was promptly treated with several antiarrhythmic medications (e.g., lidocaine 1 mg/min, esmolol 8ml/h, and nifekalant 24 ml/h after 18 ml IV injection), intravenous magnesium, electrical cardioversion, etc, but was unable to entirely control the ES. Due to suspicion of acute occlusion of the coronary artery, the cardiac team then decided to undergo emergent coronary angiography but the suggestion was refused by the patient's wife. The patient was under close monitoring in the following days; however, the ES attack remained 3–4 times each day and electrical shocks were always needed for conversion. On the fourth day of the RCA intervention, the patient's wife finally agreed to do further investigation. Repeated cardiac catheterization was then performed with the support of extracorporeal membrane oxygenation (ECMO). Coronary angiography demonstrated patency of RCA (TIMI flow grade II-III) (Figure 3C, Supplementary Video 5). Three stents were implanted in the LAD with remarkable results (TIMI flow grade III) (Figures 3D,E, Supplementary Video 6). The patient was observed to be stable overnight. However, there was another recurrence of ES on the second day (Figure 4A) after LAD intervention that remained refractory to multiple antiarrhythmic medications. Repeated TTE revealed a larger LV chamber (diameter 61 mm), worsened LVEF (15%), and severe diastolic dysfunction than that initially at admission, suggesting elevated LV filling pressure and LA pressure. We speculated that mechanical-electric feedback (MEF) may contribute to the development of ES. The cardiac team decided to create an interatrial shunt and the procedure was performed soon thereafter. After dilation with an 18 × 30 mm balloon (Figure 4B, Supplementary Video 7), the LA pressure decreased from 33 to 13 mmHg. The ES that had been present during the procedure ceased immediately (Figures 4C,D). Post-procedure TTE showed a 9 mm interatrial shunting channel (Figure 4E). The patient had no further episodes of ES during hospitalization. He received a preventative implantable cardiac defibrillator (ICD) prior to discharge and was followed up on an outpatient basis. At his last follow-up, he remained free of ES for the past 3 months and had been in good clinical condition. A review of ICD data showed no initiation of defibrillator activity. ECG revealed near sinus rhythm except for slightly elevated ST segments at the inferior leads (Figure 4F). The timeline of the patient's management during hospitalization is summarized in Table 1.

**Table 1:** Timeline of the case management.

Day 1	• Admission
	• Chest pain treated with medications
Day 8	• Uncontrolled chest pain
	• Coronary angiography
	• RCA PCI
Day 9–11	• Severe chest pain
	• Repeated ES attack, refractory to antiarrhythmic medications
Day 12	• Urgent LAD PCI under support of ECMO
Day 13	• Recurrence of ES attack
	• Interatrial shunting procedure
	• ES stops
Day 13–18	• No ES attack
Day 18	• ICD implantation
	• Withdrawal of ECMO
Day 25	• Discharge

RCA, right coronary artery; PCI, percutaneous coronary intervention; ES, electrical storm; LAD, left anterior descending artery; ECMO, extracorporeal membrane oxygenation; ICD, implantable cardiac defibrillator.

## Discussion and conclusion

Management of ES is very challenging in clinical practice. Prompt management is warranted because ES can put a patient ina life-threatening situation due to hemodynamic instability. Acute management includes the reversal of potential triggers, stabilizing patients, and therapeutic interventions to reduce the recurrence of VA. However, in clinical practice, a specific trigger of ES was not easily identified, and the situation of ES refractory to treatment approaches was often encountered. The case we presented here was a drug-refractory ES that occurred on the night of day 12 of hospitalization after percutaneous coronary intervention (PCI). ES was unable to be well-controlled despite the sequential use of antiarrhythmic drugs (lidocaine, amiodarone, esmolol, nifekalant), deep sedatives, beta-blockers, and electrical cardioversion, but it was eventually stopped by interatrial shutting procedure, which suggesting MEF as the possible pathophysiological mechanisms in this patient.

The concept of MEF as an expression of stretch-induced electrophysiological effects was first proposed by Kaufmann RL et al. where they observed stretch-induced increase in automaticity of Purkinje fibers from rhesus monkeys and transmembrane potentials under different contractile conditions on isolated cat papillary muscles (7, 8). This phenomenon obtained extensive attention thereafter and its roles in atrial and ventricular arrhythmias had been widely investigated (9–11). Mechano-electric processes are an implicit component of cardiac activity during the normal contraction of the heart, but under abnormal mechanical events, stretch-activated mechanisms may contribute to local or global changes in electrophysiology, and continuous mechanical stretch on the cardiomyocytes may be the trigger of refractory sustained ventricular arrhythmias (11, 12). Although details of these mechanisms remain complex and have yet to be fully elucidated, the following mechanisms were thought to involve the development and maintenance of ES, such as abnormal or enhanced automaticity in ventricular myocytes, early or late afterdepolarizations, micro-reentry, and transmural re-entry (13, 14) through activating several mechano-sensitive ion channels (e.g., K+-selective, Cl—selective, non-selective, and ATP-sensitive K+ channels) (6, 15).

Our patient presented to the hospital with a complaint of worsening effort angina and a coronary angiogram revealed severe three-vessel coronary artery disease (CAD). ES occurred after RCA intervention and was refractory to antiarrhythmic medications. Originally, we thought it was associated with cardiac ischemia due to suboptimal revascularization, therefore intervention on LAD was performed and successful, but ES recurred and remained refractory to multiple anti-arrhythmic medications. TTE was then repeated and showed a larger LV chamber, worse LVEF, and severe diastolic dysfunction. We speculated that mechanical stretch on cardiomyocytes due to elevated filling pressure might be the major trigger of ES based on the TTE findings. An interatrial shunting procedure was subsequently performed and confirmed our presumption. The ES stopped spontaneously along with the decrease of LA pressure from 33 to 13 mmHg, and the patient remained ES-free for up to 3 months without the use of antiarrhythmic medications.

Although the increased activation of the sympathetic nervous system was widely accepted as a major mechanism in initiating and maintaining ES (4, 5, 13), mechanically initiated ES *via* MEF should also be taken into consideration when attempted treatments were ineffective in resolving ES. In such patients, decreasing LV stretch can obtain unexpected effectiveness. This report highlights the utility of interatrial shunting as a therapeutic option in some patients with ES who are refractory to antiarrhythmic medications and there is evidence of increased left ventricular filling pressure or left atrial pressure.

## Data availability statement

The original contributions presented in the study are included in the article/Supplementary material, further inquiries can be directed to the corresponding authors.

## Ethics statement

The studies involving human participants were reviewed and approved by the Ethics Committee of Wuhan Asia Heart Hospital. The patients/participants provided their written informed consent to participate in this study.

## Author contributions

CH, RQ, LLi, MH, LLiu, and CX contributed to patient diagnosis, treatment, and follow-up. CH and LLi drafted this manuscript. LLiu and CL revised the final version of the manuscript. All authors agreed to be accountable for the content of the work.

## Conflict of interest

The authors declare that the research was conducted in the absence of any commercial or financial relationships that could be construed as a potential conflict of interest.

## Publisher's note

All claims expressed in this article are solely those of the authors and do not necessarily represent those of their affiliated organizations, or those of the publisher, the editors and the reviewers. Any product that may be evaluated in this article, or claim that may be made by its manufacturer, is not guaranteed or endorsed by the publisher.
